# Evaluation of variations of optic nerve course in relation to posterior paranasal sinuses in MDCT in a tertiary care center of Nepal: a retrospective cross-section study

**DOI:** 10.1097/MS9.0000000000001697

**Published:** 2024-01-04

**Authors:** Prajwal Dahal, Sabina Parajuli, Prajina Pradhan, Santosh Maharjan, Govinda Adhikari, Ongden Y. Tamang, Rudra P. Upadhyaya, Kapil Dawadi, Ashish Shrestha

**Affiliations:** a Department of Radiology and Imaging, Grande International Hospital; b Department of Pathology, Bir Hospital, Kathmandu, Nepal

**Keywords:** anterior clinoid process, computed tomography, optic nerve, paranasal sinuses

## Abstract

**Background:**

The proximity of optic nerves to the posterior paranasal sinuses (PNS) is a critical consideration in preventing optic nerve injuries during functional endoscopic sinus surgery.

**Methods:**

A retrospective cross-sectional study was conducted on 367 patients aged 13 years and above. Four radiologists, each with 2–6 years of experience, evaluated computed tomography scans of the PNS and the head of these patients. The optic nerves were classified into four types based on DeLano’s classification, and their respective prevalence rates were determined. Additionally, the prevalence of optic nerve dehiscence and pneumatization of the anterior clinoid process was assessed.

**Results:**

A total of 734 optic nerves were evaluated and categorized into four groups as per DeLano’s classification. The most common type was classified as type 1 optic nerve, representing 65.4% of all optic nerves. Types 2, 3, and 4 optic nerves accounted for 16.9, 8.6, and 9.1%, respectively. The prevalence rates of type 1, type 2, type 3, and type 4 optic nerves were 76.6, 24.5, 12.3, and 14.4%, respectively. Optic nerve dehiscence was observed in 14.3% of cases, with a prevalence of 21.2% in the study. Type 3 optic nerves were most commonly associated with dehiscence, with 49.2% of them showing dehiscence in their course through the ethmoid or sphenoid sinus. The prevalence of pneumatization of the anterior clinoid process in the study population was 28.3%, with type 3 optic nerves being the most frequently associated.

**Conclusion:**

An understanding of the relationship between optic nerves and posterior PNS, as well as awareness of optic nerve dehiscence, is essential in preventing injuries during sinus surgeries. Type 1 optic nerve predominates among Nepalese patients visiting our hospital.

## Introduction

Highlights

- This is the first study assessing optic nerve types, dehiscence, and anterior clinoid processes in Nepalese.
- Frequency of type 1 optic nerves: 65.4%; type 2: 16.9%; type 3: 9.1%; type 4: 8.6%. Prevalence: type 1 (76.6%), type 2 (24.5%), type 3 (12.3%), type 4 (14.4%). Type 4 being more common than type 3.
- Frequency of optic nerve dehiscence: 14.3%; type 3 most affected; overall prevalence: 21.3%.
- Prevalence of pneumatized anterior clinoid process: 28.3%; highest association with type 3 optic nerves.

Optic nerve is the second cranial nerve. It originates from the eye and courses toward the middle cranial fossa in a bony optic canal. It meets the optic nerve of opposite side in cranium to form the optic chiasma^1^. Within the optic canal, it closely associates with the posterior sinuses, particularly the posterior ethmoidal cells and the sphenoid sinus^2^. The relationship between the optic nerve and these sinuses depends on the extent of sinus pneumatization. Typically, the optic nerve is situated above and to the side of the sphenoid sinus^3^. As the number of functional endoscopic sinus surgeries (FESS) performed annually increases, so do the complications associated with the procedure, including the risk of optic nerve injury^4^.

Of significant surgical relevance is the Onodi cell, as described by Rice, Schaefer, and Lang^2^. The anatomical terminology group has defined Onodi cell as the rearmost ethmoid air cell situated superiorly and laterally to the sphenoid sinus^5^. When there is pneumatization of ethmoid air cells above and to the side of the sphenoid sinus, the optic nerve comes into close proximity to the ethmoid and sphenoid sinuses, and this specific posterior ethmoid cell is referred to as the Onodi cell^2^. The presence of Onodi cells increases the susceptibility to optic nerve injury during FESS^4^. Moreover, the optic nerve may sometimes lack a thin bony covering, further elevating the risk of injury during FESS^4^.

The degree of pneumatization of paranasal sinuses (PNS) exhibits variation based on ethnic background. Our study aims to ascertain the prevalence of different types of optic nerves according to DeLano’s classification^6^, the occurrence of optic nerve dehiscence, and the prevalence of pneumatization of the anterior clinoid process within the Nepalese population, which comprises individuals of both Indo-Aryan and Mongolian descent.

## Material and methods

A retrospective cross-sectional study was carried out in the department of radiodiagnosis and imaging of a private tertiary care center located in Nepal, spanning from January 2022 to May 2023. Ethical clearance for the study was secured from the institutional review committee. Patient consent requirements were waived by the institutional review committee, given that the study was retrospective and did not involve any active interventions.

The primary aim and objectives of our study were to analyze variations of optic nerves in relation to posterior PNS, categorize the optic nerves as per modified DeLano’s classification^2^ and find out the prevalences of different types of optic nerves. Similarly, we aim to find out the prevalences of dehiscence of optic nerve and pneumatization of anterior clinoid process in Nepalese population visiting our hospital.

The sample size for the study was determined using the following formula:


$$n=Z^{2}\;P\;\left(1-P\right)/d^{2}.$$

Where:

Z- 2.576 (for 99% CI).

P- Frequency of most common anatomical variant from previous study (60%)^2^.

d- margin of error (5%).

According to the formula mentioned above, the minimum required optic nerves for the study was 637. Since each computed tomography (CT) scan evaluated two optic nerves, 319 CT scans were required for the study. However, our study involved the analysis of 367 plain CT scans of the head, which were referred for issues related to headache and dizziness, as well as PNS scans referred for chronic rhinosinusitis in patients aged 13 years and older. We have retrospectively analyzed the noncontrast CT of head and PNS done for treatment purpose. The patients were not exposed to radiation for the purpose of research. Average radiation exposure during this investigation was 1.5–3 mSv^2^.We excluded CT scans of the head and PNS that exhibited trauma, significant lesions, a history of previous surgical procedures, or extensive polyposis. Additionally, we excluded CT scans of patients under the age of 13 since their sphenoid sinus pneumatization is still incomplete. The work has been reported in line with the Strengthening the Reporting of Cohort, Cross-sectional and Case–control Studies in Surgery (STROCSS) criteria^7^.

The evaluation of these CT scans was carried out through a random selection process. Four radiologists, each with 2–6 years of experience, independently interpreted the CT scans. In cases where there was confusion or uncertainty, the radiologists engaged in mutual consultation to arrive at a consensus. The CT scans were acquired using a 160-slice MDCT scanner in helical mode, featuring a slice thickness of 0.65 mm, 120 kV, and auto mAs settings. Volumetric data from the scans were acquired and then reformatted into axial, coronal, and sagittal planes for analysis, all of which was conducted on a specialized workstation.

DeLano classified optic nerves into four types^6^ based on relationship with the posterior PNS. We slightly modified the DeLano’s classification for better objective classification as in the study by Itagi *et al*.^2^. The optic nerves were classified into following four types based on the modified DeLano’s classification^2^ (Table 1).

**Table 1:** Modified DeLano *et al*. classification^2^ of optic nerve

Type of optic nerve	Criteria
Type 1	Optic nerve supero-lateral to sphenoid sinus with no indentation in sphenoid sinus. [Fig. 1]
Type 2	Optic nerve causes indentation on sphenoid sinus (<50% circumference). [Fig. 2]
Type 3	Optic nerve causes indentation on sphenoid sinus (≥50% circumference). [Fig. 3]
Type 4	Optic nerve is lateral to ethmoid and sphenoid sinuses. [Fig. 4]

**Figure 5:**
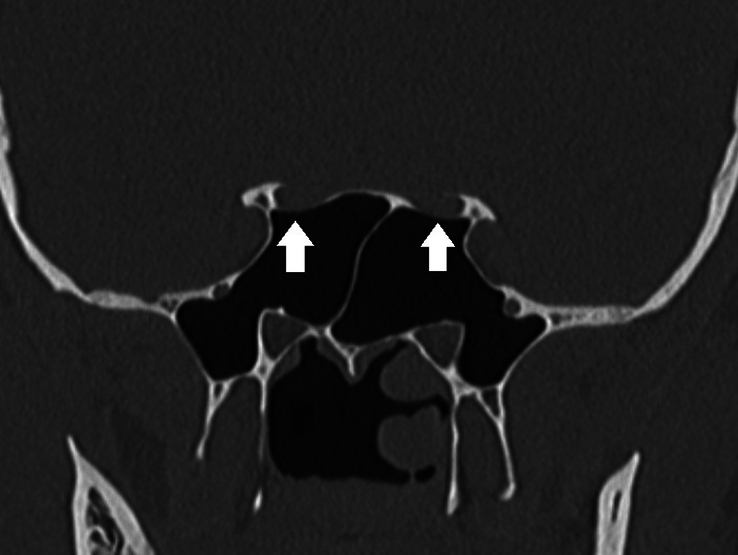
There is absence of bony covering of bilateral optic nerves in sphenoid sinus (white arrows). Note type 3 course of bilateral optic nerves.

**Figure 6:**
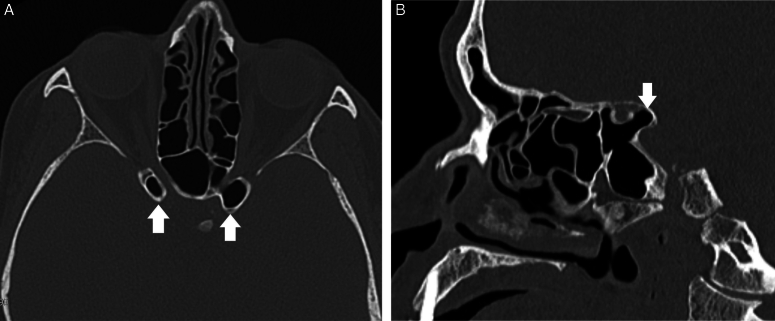
Fig. 6A is an axial CT image of PNS at the level of optic nerves. Figure 6B is mid sagittal CT image. Pneumatized anterior clinoid process is seen bilaterally in both planes (white arrows).

The optic nerves were evaluated in all planes; however, the classification was done in coronal plane. Dehiscence of the optic nerve was defined as the absence of bony covering observed in coronal bone window images when the optic nerve passes in close proximity to the ethmoid and sphenoid sinuses^8^ [Fig. 5]. Pneumatization of the anterior clinoid process was defined as the extension of sphenoid sinus pneumatization into the anterior clinoid process^9^ [Fig. 6A, 6B]. The obtained data were entered into Excel 2019 and analyzed using IBM SPSS. The χ^2^ test was employed for correlation and *P*-value <0.05 was considered significant.

## Result

A total of 734 optic nerves were assessed in 367 CT scans of the PNS and the head, which included CT scans of 171 males and 196 females aged 13–91 years. The CT scans were evaluated for the relationship of optic nerves with posterior PNS and dehiscence of optic nerves. Additionally, an assessment of the pneumatization of anterior clinoid processes was conducted. The prevalence of different types of optic nerves, dehiscence of optic nerves, and pneumatization of anterior clinoid process were obtained. The frequency and prevalence of different types of optic nerves in our study is presented in Table 2.

**Table 2:** Frequency and prevalence of different types of optic nerves

Type of optic nerve	Right	Left	Both sides	Total optic nerves	Frequencies of optic nerves	Prevalence of optic nerves
Type 1	239	241	199	480	65.4%	76.6%
Type 2	62	62	34	124	16.9%	24.5%
Type 3	31	32	18	63	8.6%	12.3%
Type 4	35	32	14	67	9.1%	14.4%

The most common optic nerve course was type 1 followed by type 2 course. Type 4 course was more prevalent than type 3 (Table 2). The same type of optic nerves was observed on both sides in 72.2% subjects. Among these subjects, type 1 optic nerves were seen on both sides in 75.1%; type 2, type 3, and type 4 optic nerves were seen on both sides in 12.8, 6.8, and 5.3% subjects, respectively. In the remaining 17.8% subjects, different types of optic nerves were observed on the two sides.

Dehiscence was defined as the absence of bony covering along the course of the optic nerves. Dehiscence was observed in 105 optic nerves (14.3%) of 78 individuals. Of these 78 individuals, 41 were female, and 37 were male. There was no sex predilection for dehiscence of optic nerve. The prevalence of optic nerve dehiscence in our study was 21.3%. Dehiscence was observed in 49 optic nerves on the right side and 56 optic nerves on the left side. In 27 cases, there was dehiscence of bilateral optic nerves. The frequency of bony dehiscence was calculated for each type of optic nerve. The most common type of optic nerve associated with dehiscence was type 3 followed by type 4 (Table 3).

**Table 3:** Dehiscence and pneumatization of anterior clinoid process (PACP) associated with different types of optic nerves

Type of optic nerve	Number of canals	Bone dehiscence	% Of Bone dehiscence	PACP	% of PACP
Type 1	480	28	5.8	42	8.75
Type 2	124	17	13.7	46	37.1
Type 3	63	31	49.2	44	69.8
Type 4	67	29	43.2	25	37.3
Total	734	105	14.3	157	21.4

Similarly, pneumatization of anterior clinoid process was observed in 104 subjects. It was observed on the right side in 78 (75%) subjects and on the left side in 79 (75.9%) subjects. It was observed bilaterally in 53 (50.9%) subjects. The prevalence of pneumatization of anterior clinoid process was 28.3% in our study. A total of 157 optic nerves (20.9%) were associated with pneumatization of anterior clinoid process. In our study, type 3 and type 4 optic nerves were most frequently associated with pneumatization of anterior clinoid process (Table 3). The association of pneumatization of anterior clinoid process was statistically significant in type 3 optic nerves, with a *P*-value <0.05.

## Discussion

The sphenoid sinus is typically present from birth, and its pneumatization continues until around the age of 13^10^. Situated centrally in the skull base, variations in the pneumatization of the sphenoid sinus and posterior ethmoid sinuses can affect the relationship between the optic nerve and these structures^11^. Medical CT scans of the head and PNS are commonly performed in specialized medical centers to assess conditions such as headaches, dizziness, trauma, chronic rhinosinusitis, and PNS polyposis. These scans provide detailed views of the paranasal sinus anatomy and adjacent structures, including the optic nerves^12^. Understanding the course of the optic nerve is crucial for preventing injuries during FESS. Having a bony covering around the optic nerve offers protection against potential injuries and the spread of infections from adjacent sinuses^2^. Pneumatization of anterior clinoid process represents a form of extensive pneumatization of the sphenoid sinus. Pneumatization of anterior clinoid process can displace the optic nerve medially, causing it to indent into the sphenoid and ethmoid sinuses^9^.

Numerous studies have been conducted in different populations to determine the prevalence of various optic nerve types, optic nerve dehiscence, and the presence of the anterior clinoid process. In nearly all of these studies, type 1 optic nerves are the most common, with prevalence rates ranging from 55.9 to 83.3%. Details of these studies and their findings are summarized in Table 4.

**Table 4:** Prevalences of different types of optic nerves, dehiscence of optic nerves and PACP obtained in various studies conducted worldwide

References	Study population	Sample size	Frequency/Prevalence of type 1 optic nerve	Frequency/Prevalence of type 2 optic nerve	Frequency/Prevalence of type 3 optic nerve	Frequency/Prevalence of type 4 optic nerve	Frequency/Prevalence of optic nerve dehiscence	Frequency/Prevalence of PACP
Sapçi *et al*.^13^	Turkey	100					13.5%	11%
Heskova *et al*.^14^	Slovakia	34	55.9%	14.7%	23.5%	5.9%	11.8%	26.5%
BN *et al*.^15^	Karnataka (India)	124						25.1%
B I *et al*.^3^	Kerela (India)	336	62.6%	18.6%	9.6%	9.6%	9.8%	11.2%
Itagi *et al*.^2^	Banglore (India)	100	60%	15%	14%	11%	17.5%	15%
Al-Tameemi and Hak^16^	Iraq	100					8.5%	4.5%
Dias *et al*.^12^	Brazil	203	78.9%	16.8%	3.47%	0.74%	21.29%	10.6%
DeLano *et al*.^6^	USA	150					24%	4%

Based on the literatures reviewed above, it is evident that the prevalence of different types of optic nerves varies among different populations. Type 1 optic nerve is consistently the most common type in nearly all studies, with prevalence ranging from 55.9 to 83.3%. Type 2 and type 3 optic nerves, with prevalence rates ranging from 8.9 to 26.8% and 3.47 to 23.5%, respectively, are the second and third most common types in most of these studies. Type 4 optic nerve is consistently the least common, with prevalence rates ranging from 0.74 to 21.7%.

In our study, type 1 optic nerve is also the most common, with a prevalence of 76.5%. Type 2, type 3, and type 4 optic nerves are observed in 24.5, 12.2, and 14.4% of cases, respectively. Interestingly, in contrast to the findings of most studies, type 3 optic nerve is the least common in our study.

The prevalence of dehiscent optic nerves has been reported to range from 8.5 to 24% in previous studies. Our results show a prevalence of 21.2%, which is consistent with previous findings. Similarly, the prevalence of pneumatization of anterior clinoid process has been reported to range from 4 to 26.5% in available literature. In our study, we found a prevalence of 27.8%, slightly higher than in previous studies. Since prevalence of type 3 optic nerve, type 4 optic nerve, dehiscent optic nerves and PACP are higher in our study population, chances of optic nerve injury are high during FESS. Knowledge of anatomy of PNS, relationship of optic nerves with posterior PNS is a must for surgeons before FESS. Precautions should be taken to prevent injury of optic nerves during FESS in our study population.

The most significant strength of our study is the sample size. We have obtained an adequate sample size, and to the best of our knowledge, we could not find other similar studies with a comparable sample size conducted in the Nepalese population. Another strength of our study lies in the analysis of CT scans by four highly experienced radiologists. Any ambiguous findings were thoroughly discussed among the radiologists, leading to logical conclusions.

While we acknowledge that we have achieved an adequate sample size based on standard statistical equations, we believe that larger-scale studies encompassing samples from various regions of Nepal are necessary to provide a comprehensive representation of the Nepalese population. Furthermore, the CT were read by four radiologists with different level of experiences. This could result in observer bias. This is another limitation of our study. Nevertheless, our study can serve as a valuable and representative contribution to the understanding of this population.

## Conclusion

**Figure 1:**
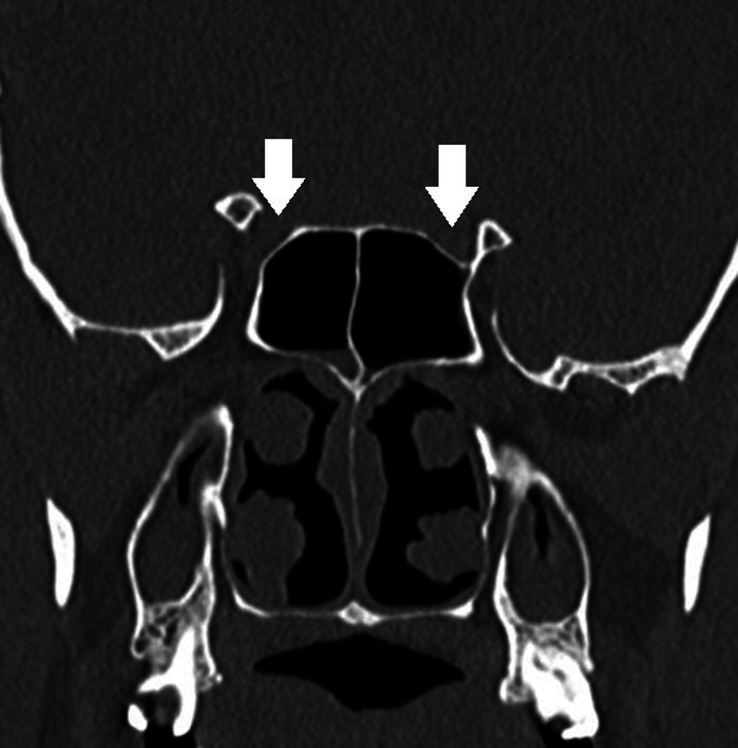
DeLano type 1 course of bilateral optic nerves. The optic nerves course supero-lateral to sphenoid sinus (white arrows).

**Figure 2:**
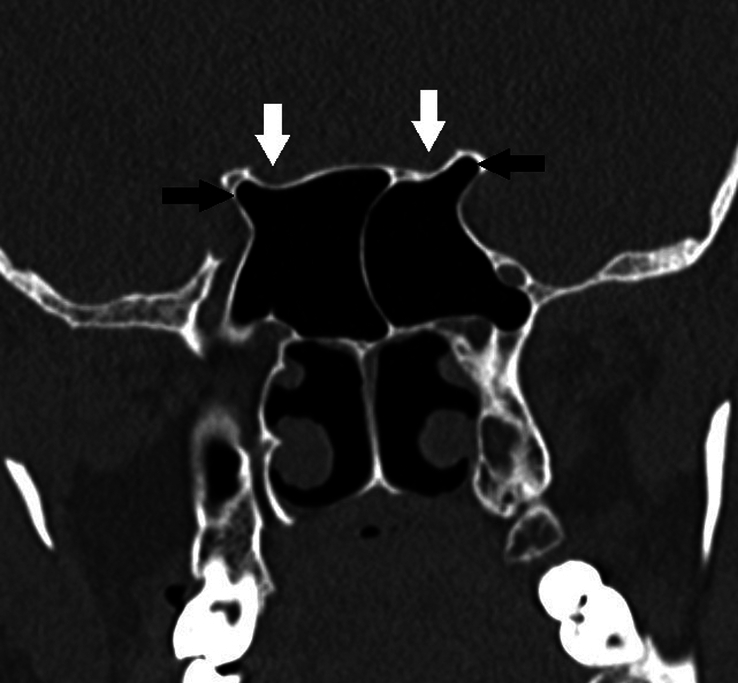
Modified DeLano type 2 course of bilateral optic nerves. There is indentation of <50% circumference of bilateral optic nerves into the sphenoid sinus (white arrows). Also, note bilateral pneumatized anterior clinoid processes (black arrows).

**Figure 3:**
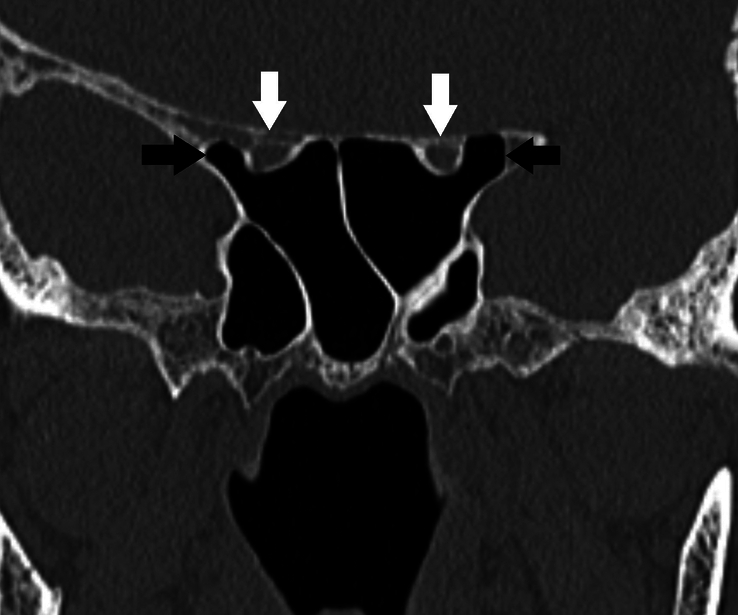
Modified DeLano type 3 course of bilateral optic nerves. There is indentation of >50% circumference of bilateral optic nerves into the sphenoid sinus (white arrows). Also, note bilateral pneumatized anterior clinoid processes (black arrows).

**Figure 4:**
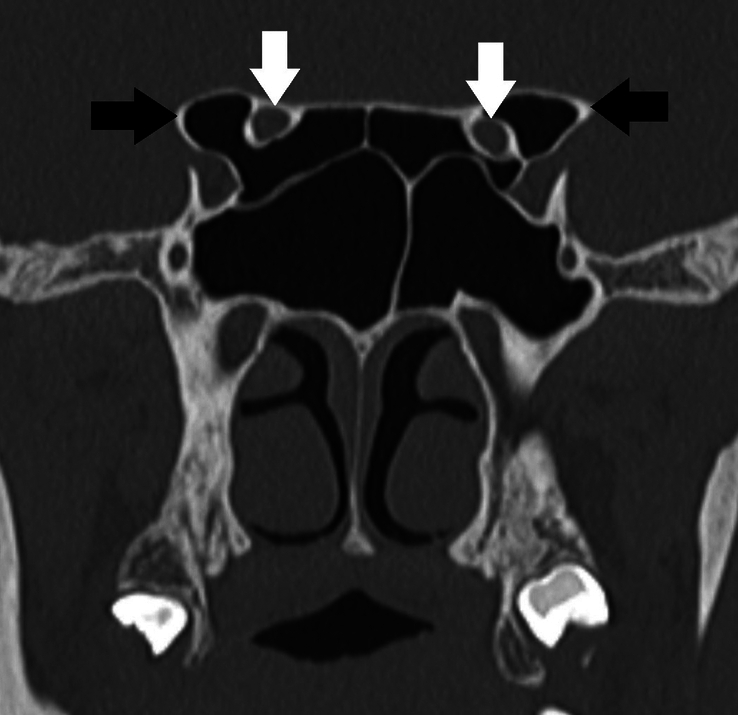
DeLano type 4 course of bilateral optic nerves. The posterior ethmoid air cells extend supero-lateral to the sphenoid sinus. The right optic nerve courses superior to the posterior ethmoid air cell and the left optic nerve courses through the posterior ethmoid air cell (white arrows). Also, note bilateral pneumatized anterior clinoid processes (black arrows).

The vulnerability of the optic nerve to injury increases significantly during FESS, particularly in cases involving type 3 and type 4 optic nerves. When there is a dehiscence or weakening of the optic nerve, the risk of injury becomes even more pronounced. In our study, we found that type 1 optic nerves are the most prevalent in our study population, with type 4 optic nerves being more common than type 3. There is significant association of pneumatization of anterior clinoid process and type 3 optic nerve (Figs 1–4).

## Ethical approval and consent to participate

Ethical approval has been obtained from institutional review committee Grande International.

Hospital, Tokha, Kathmandu. Reference number of the IRC letter: 16/2023.

## Consent for publication

The consent was waived by the institutional review committee since the study was retrospective in nature.

## Sources of funding

The research did not receive any specific grant from any funding agency in the public, commercial or non-profit sector.

## Author contribution

P.D.: supervision, writing the manuscript, and software; S.P.: edit manuscript and data curation; P.P., G.A., and S.M.: statistical analysis; O.Y.T.: data curation; R.P.U., K.D., and A.S.: data curation.

## Conflicts of interest disclosure

The authors declare that they have no known competing financial interests or personal relationships that could have appeared to influence the work reported in this paper.

## Research registration unique identifying number (UIN)

NCT06077929.

## Guarantor

The principal author Prajwal Dahal accepts full responsibility for the work and conduct of the study. I have access to the data and control the decision to publish.

## Availability of data and material

Data sharing is not applicable to this article as no datasets were generated or analyzed during the current study.

## Provenance and peer review

Paper was not invited.
